# Supplementary material for: Content-rich biological network constructed by mining PubMed abstracts
Source: BMC Bioinformatics. 2004 Oct 8;5:147. doi: 10.1186/1471-2105-5-147 (PMC528731; doi:10.1186/1471-2105-5-147)
Supplement: Additional File 2 — The original results of the above study (non-essential files are deleted to keep the file size under the limit set by BMC bioinformatics). [file 1471-2105-5-147-S2.bz2 › chilibotAdditionalFile2/dip05/612ID7791872E10995/html/RAF1_RAP1A.html]

 


 **RAF1** and **RAP1A** 
  
Found 29 abstracts in PubMed, retrieved 05.  
 

 What does Google say? 
 PDF only 
| .edu only 

---

**Interactive relationship** (e.g. stimulation, inhibition, etc)

**Inhibitory relationship**- The effect of  **Rap1A**  V12 was dramatically potentiated by the concurrent activation of PKC, the cAMP pathway, and Ras, and this potentiation was blocked by dominant negative mutants of Ras and Raf  [ **RAF1** ] .  Ref: 12473665 J Biol Chem, 2003
**Neutral relationship**

**Non-interactive relationship** (e.g. studied together, co-existance, homology, etc.)

- In cells expressing the activated  **Rap1A**  protein, a PI3K Raf  [ **RAF1** ]  MEK dependent pathway mediates transient ERK activation.  Ref: 11146560 Oncogene, 2000
